# Clinical significance of *Chlamydia trachomatis* and HPV coinfection across cervical lesion severity: a 10-year retrospective study in Southern China

**DOI:** 10.3389/fcimb.2026.1832343

**Published:** 2026-09-11

**Authors:** Huan Li, Hongyu Zhao, Lihua Liang, Minchai Chen, Xiaohan Yang

**Affiliations:** 1 Department of Clinical Laboratory, Guangdong Women and Children Hospital, Guangzhou, China; 2 Women and Children’s Hospital, Southern University of Science and Technology, Guangzhou, China; 3 Medical Genetic Center, Guangdong Women and Children Hospital, Guangzhou, China

**Keywords:** cervical lesions, *Chlamydia trachomatis*, coinfection, DNA methylation, human papillomavirus

## Abstract

**Background:**

Persistent high-risk HPV infection is the major cause of cervical carcinogenesis, but the clinical relevance of *Chlamydia trachomatis* (CT) coinfection remains uncertain. We examined the epidemiological patterns of CT–HPV coinfection and their associations with cervical lesion severity among women in Southern China.

**Methods:**

This retrospective study included 21,275 outpatients who underwent concurrent HPV genotyping, CT PCR testing, and cytological or histopathological evaluation between 2015 and 2024. Age-specific patterns and temporal trends were assessed. Age-adjusted logistic regression was used to examine associations of HPV and CT with cervical lesion categories. Six DNA methylation markers were analyzed in a subset of samples.

**Results:**

Overall prevalence was 25.7% for HPV, 8.7% for CT, and 3.8% for HPV–CT coinfection, with declining trends over time. CT infection and HPV–CT coinfection were more common in younger women. HPV positivity was significantly higher in CT-positive than CT-negative women (43.9% vs 23.9%, *p* < 0.001). CT infection and coinfection were more frequently observed in ASC-US/-H and LSIL, while CT showed weaker associations with advanced lesions and was not significantly associated with invasive cervical carcinoma. Among the six methylation markers, only PAX1 showed significant differences among the three groups, while HPV–CT coinfection was not associated with additional methylation alterations compared with HPV infection alone.

**Conclusions:**

CT infection and HPV–CT coinfection were concentrated in younger women and were more frequently associated with low-grade cervical abnormalities. CT positivity was also associated with a higher prevalence of HPV infection.

## 1 Introduction

Cervical cancer remains a major public health problem, particularly in low- and middle-income countries (LMICs) where organized screening and access to human papillomavirus (HPV) vaccination are limited. GLOBOCAN 2022 estimated 662,300 new cases and 348,900 deaths worldwide, ranking cervical cancer fourth for incidence and mortality among women (Bray et al., 2024). More than 90% of the burden occurs in LMICs, reflecting gaps in prevention and early detection.

Persistent infection with high-risk HPV (HR-HPV) underlies cervical carcinogenesis, and HR-HPV DNA is detected in more than 95% of invasive tumors (Cancer Genome Atlas Research N et al., 2017). The viral oncoproteins E6 and E7 disable the p53 and retinoblastoma pathways, promoting genome destabilization and loss of cell-cycle checkpoints (Letafati et al., 2024; Blanco and Munoz, 2025). Acquisition of HR-HPV alone, however, seldom leads to high-grade intraepithelial lesions (HSIL) or invasive disease. Most infections are transient and cleared by host immunity; only about 10 to 20% persist, and roughly 12% of those persistent infections progress to precancerous or malignant outcomes (Munoz-Bello et al., 2022; Zhao et al., 2024). Progression risk is further shaped by cofactors such as immunosuppression, smoking, hormonal exposures, and coinfections with other sexually transmitted infections (Choi et al., 2023; Hewavisenti et al., 2023; Blanco and Munoz, 2025).

Among these potential cofactors for HR-HPV persistence and cervical lesion progression, *Chlamydia trachomatis* (CT) is one of the most common bacterial sexually transmitted infections worldwide. Although often asymptomatic, CT infection can induce chronic inflammation, disrupt epithelial integrity, and modulate local immune responses, thereby creating a microenvironment that may facilitate HPV persistence (Safaeian et al., 2010; Ji et al., 2019; Lu et al., 2024). Epidemiological studies have reported higher HPV prevalence and increased risks of cervical intraepithelial neoplasia (CIN) among CT-positive women, whereas other studies have found no independent association between CT infection and cervical premalignancy (Safaeian et al., 2010; Zhu et al., 2016; Ji et al., 2019). These inconsistent findings suggest that the clinical significance of HPV–CT coinfection, particularly its association with cervical lesion severity, remains unclear.

Host DNA methylation alterations are increasingly recognized as molecular features of HPV-associated cervical carcinogenesis. Methylation of genes such as paired box 1 (PAX1) has been associated with high-grade cervical lesions and has shown potential value in the triage of HPV-positive women (Lai et al., 2008; Kong et al., 2015; Chen et al., 2024). Other markers, including family with sequence similarity 19 member A4 (FAM19A4) and erythrocyte membrane protein band 4.1-like 3 (EPB41L3), have also been investigated as indicators of cervical transformation (Molano et al., 2024). However, whether concurrent CT infection is associated with additional methylation alterations beyond those observed with HPV infection alone remains unclear.

The clinical significance of HPV–CT coinfection therefore remains incompletely defined, particularly across different stages of cervical disease. We conducted a ten-year retrospective study of women attending a tertiary women’s and children’s hospital in Southern China to examine the prevalence, age distribution, and clinicopathological patterns of HPV infection, CT infection, and HPV–CT coinfection. We also evaluated selected DNA methylation markers to determine whether CT coinfection was associated with additional epigenetic alterations in HPV-positive women.

## 2 Materials and methods

### 2.1 Study design and participants

This retrospective study included 21,275 women aged ≥18 years who underwent cervical assessment at Guangdong Women and Children Hospital (Guangzhou, China) from January 2015 to December 2024. Participants were identified from routine clinical testing records. Eligible women had concurrent HPV and CT testing and cytological evaluation, with colposcopy-guided histopathological assessment performed when clinically indicated, and had complete demographic and clinical data available. Women were excluded if they were pregnant at sampling, had undergone total hysterectomy, presented with systemic infection or autoimmune disease, underwent uterine surgery within the previous week, or had a malignancy other than cervical cancer. In addition, an exploratory DNA methylation substudy was performed in a subset of participants using residual cervical exfoliated-cell specimens. Ethical approval was obtained from the hospital’s Ethics Committee.

### 2.2 Cytological and histopathological classification

Cytology was performed using the ThinPrep cytologic test (TCT) and categorized under the 2014 Bethesda System (Nayar and Wilbur, 2015) as: negative for intraepithelial lesion or malignancy (NILM), inflammatory changes, atypical squamous cells of undetermined significance or atypical squamous cells, cannot exclude HSIL (ASC-US/-H), low-grade squamous intraepithelial lesion (LSIL), HSIL, and invasive cervical carcinoma (ICC). Histopathological examination was performed in selected patients when further diagnostic evaluation was considered clinically necessary by the treating physician, particularly in the presence of abnormal cytological or colposcopic findings. For analysis, histopathological diagnoses of CIN1 were categorized as LSIL, CIN2/3 as HSIL, and invasive malignancy as ICC. When histopathological results were available, they were used as the final diagnostic classification.

### 2.3 HPV genotyping

HPV DNA was detected and genotyped with a commercial assay (Kaipu Biotechnology, Guangzhou, China) based on PCR amplification of the L1 consensus region followed by flow-through hybridization. The assay identifies 21 HPV genotypes, including 15 genotypes classified as high-risk by the manufacturer (HPV16, 18, 31, 33, 35, 39, 45, 51, 52, 53, 56, 58, 59, 66, and 68) and six low-risk genotypes (HPV6, 11, 42, 43, 44, and CP8304). HPV53 was therefore included in the HR-HPV group in the present analysis in accordance with the manufacturer’s classification. Each run included positive and negative controls for quality assurance, and procedures were performed according to the manufacturer’s instructions, as described previously (Yang et al., 2023).

### 2.4 Detection and quantification of CT

A separate cervical swab was collected for CT DNA testing during the same clinical visit as HPV testing and cytological assessment. CT DNA was quantified using a real-time PCR assay (DaAn Gene, Guangzhou, China) targeting the cryptic plasmid. Cervical swabs were processed per manufacturer’s instructions. Samples with no amplification curve or undetermined Ct were classified as below detection. For Ct < 30, copy numbers (C) were derived: C < 5.0 × 10^2^ copies/mL was reported as < 5.0 × 10^2^ copies/mL; 5.0 × 10^2^ ≤ C ≤ 5.0 × 10^8^ copies/mL was reported as the measured value; C > 5.0 × 10^8^ copies/mL was retested after dilution.

### 2.5 DNA methylation testing

DNA methylation analysis was conducted as an exploratory substudy using residual cervical exfoliated-cell specimens. A total of 45 samples were included, comprising 16 HPV–CT coinfected cases, 19 HPV-positive/CT-negative cases, and 10 controls.

Genomic DNA was extracted, bisulfite-converted, and purified using a magnetic bead-based pretreatment kit (Wuhan Kindwise, China) according to the manufacturer’s instructions. DNA yield and purity were evaluated using a NanoDrop 2000c spectrophotometer (Thermo Fisher Scientific, USA).

DNA methylation was analyzed using three commercial kits approved by the National Medical Products Administration (NMPA). The PAX1/JAM3 and CDO1/CELF4 kits (OriginPoly, Beijing, China) targeted PAX1, junctional adhesion molecule 3 (JAM3), cysteine dioxygenase type 1 (CDO1), and CUGBP Elav-like family member 4 (CELF4) (Qi et al., 2023; Chen et al., 2024), whereas the AJAP1/GALR1 kit (Wuhan Kindwise, Wuhan, China) targeted adherens junctions-associated protein 1 (AJAP1) and galanin receptor 1 (GALR1). PCR was performed on the SLAN-96S platform. Glyceraldehyde-3-phosphate dehydrogenase (GAPDH) served as the internal reference gene for the PAX1/JAM3 and CDO1/CELF4 assays, whereas β2-microglobulin (B2M) served as the internal reference gene for the AJAP1/GALR1 assay. Methylation was quantified as ΔCt = Ct_target − Ct_control, where Ct_target represents the cycle threshold of the methylation target gene and Ct_control represents the cycle threshold of the corresponding internal reference gene. A lower ΔCt value indicates a higher relative methylation level.

### 2.6 Statistical analysis

HPV, CT, and coinfection prevalence was calculated for each cytological and histopathological category and presented with 95% confidence intervals (95% CI). Temporal changes in prevalence from 2015 to 2024, stratified by HPV genotype, were assessed using the Linear-by-Linear Association Chi-square Test. Comparisons of categorical data were performed with the *χ*^2^ test or Fisher’s exact test, and continuous variables with the Student’s *t*-test or Mann–Whitney U test. Associations between infection status (CT, HPV, or HPV–CT coinfection) and cervical lesions (ASC-US/-H, LSIL, HSIL, ICC) were evaluated using multivariable logistic regression adjusted by age, with results expressed as odds ratios (ORs) and 95% CIs. Analyses were carried out in R version 4.3.1 using the *stats* and *coin* packages. For methylation data, normality and homogeneity of variance were assessed before group comparisons. Normally distributed data were compared using one-way ANOVA followed by Tukey’s *post-hoc* test; non-normally distributed data were analyzed using the Kruskal–Wallis test followed by Dunn’s *post-hoc* test with adjustment for multiple comparisons. A two-tailed *p* value < 0.05 was considered significant.

## 3 Results

### 3.1 Prevalence and temporal trends of HPV and CT infections

Among 21,275 women, the overall prevalence of HPV, CT, and HPV–CT coinfection was 25.7%, 8.7%, and 3.8%, respectively. HR-HPV was markedly more common than LR-HPV (21.1% vs. 9.0%, *p* < 0.001). The most frequent HR-HPV genotypes were HPV52 (6.5%), HPV16 (3.3%), HPV58 (3.2%), and HPV51 (2.7%), while HPV6 (3.0%) and HPV11 (1.6%) predominated among LR-HPV (**Table 1**). Of all participants, 17.1% carried single HPV infections and 8.6% harbored multiple infections (**Supplementary Table 1**).

**Table 1:** Longitudinal trends in the prevalence of CT, HPV, and HPV–CT coinfection, with genotype-specific HPV distribution from 2015 to 2024.

Variable	Overall (n=21275)	Prevalence by year.									
		2015 (n=1643)	2016 (n=2024)	2017 (n=2202)	2018 (n=2374)	2019 (n=2431)	2020 (n=1974)	2021 (n=2285	2022 (n=2322)	2023 (n=2493)	2024 (n=1527)
CT	1859 (8.7)	190 (11.6)	207 (10.2)	232 (10.5)	230 (9.7)	209 (8.6)	173 (8.8)	178 (7.8)	179 (7.7)	166 (6.7)	95 (6.2)
HPV (any type)	5467 (25.7)	488 (29.7)	525 (25.9)	595 (27.0)	613 (25.8)	617 (25.4)	525 (26.6)	603 (26.4)	532 (22.9)	601 (24.1)	368 (24.1)
HPV–CT coinfection	817 (3.8)	83 (5.1)	79 (3.9)	99 (4.5)	113 (4.8)	96 (3.9)	91 (4.6)	76 (3.3)	67 (2.9)	72 (2.9)	41 (2.7)
LR-HPV (any type)	1915 (9.0)	173 (10.5)	198 (9.8)	215 (9.8)	211 (8.9)	200 (8.2)	195 (9.9)	212 (9.3)	192 (8.3)	194 (7.8)	125 (8.2)
HPV6	647 (3.0)	66 (4.0)	81 (4.0)	69 (3.1)	60 (2.5)	76 (3.1)	73 (3.7)	64 (2.8)	56 (2.4)	68 (2.7)	34 (2.2)
HPV11	338 (1.6)	53 (3.2)	31 (1.5)	52 (2.4)	29 (1.2)	34 (1.4)	32 (1.6)	38 (1.7)	27 (1.2)	20 (0.8)	22 (1.4)
HPV42	310 (1.5)	0 (0.0)	4 (0.2)	24 (1.1)	48 (2.0)	47 (1.9)	38 (1.9)	46 (2.0)	39 (1.7)	35 (1.4)	29 (1.9)
HPV43	227 (1.1)	1 (0.1)	3 (0.1)	22 (1.0)	34 (1.4)	27 (1.1)	31 (1.6)	29 (1.3)	29 (1.2)	30 (1.2)	21 (1.4)
HPV44	220 (1.0)	6 (0.4)	11 (0.5)	20 (0.9)	28 (1.2)	18 (0.7)	32 (1.6)	27 (1.2)	29 (1.2)	31 (1.2)	18 (1.2)
CP8304	494 (2.3)	69 (4.2)	81 (4.0)	55 (2.5)	50 (2.1)	39 (1.6)	41 (2.1)	45 (2.0)	39 (1.7)	43 (1.7)	32 (2.1)
HR-HPV (any type)	4488 (21.1)	410 (25.0)	434 (21.4)	496 (22.5)	498 (21.0)	525 (21.6)	406 (20.6)	498 (21.8)	429 (18.5)	493 (19.8)	299 (19.6)
HPV16	699 (3.3)	69 (4.2)	73 (3.6)	83 (3.8)	82 (3.5)	74 (3.0)	82 (4.2)	90 (3.9)	58 (2.5)	55 (2.2)	33 (2.2)
HPV18	300 (1.4)	32 (1.9)	38 (1.9)	40 (1.8)	33 (1.4)	37 (1.5)	24 (1.2)	30 (1.3)	18 (0.8)	32 (1.3)	16 (1.0)
HPV31	165 (0.8)	15 (0.9)	20 (1.0)	17 (0.8)	18 (0.8)	25 (1.0)	13 (0.7)	20 (0.9)	16 (0.7)	14 (0.6)	7 (0.5)
HPV33	222 (1.0)	20 (1.2)	33 (1.6)	29 (1.3)	20 (0.8)	28 (1.2)	23 (1.2)	18 (0.8)	19 (0.8)	17 (0.7)	15 (1.0)
HPV35	86 (0.4)	5 (0.3)	9 (0.4)	10 (0.5)	13 (0.5)	10 (0.4)	8 (0.4)	9 (0.4)	11 (0.5)	5 (0.2)	6 (0.4)
HPV39	464 (2.2)	40 (2.4)	51 (2.5)	45 (2.0)	49 (2.1)	66 (2.7)	46 (2.3)	55 (2.4)	40 (1.7)	40 (1.6)	32 (2.1)
HPV45	73 (0.3)	5 (0.3)	7 (0.3)	5 (0.2)	6 (0.3)	13 (0.5)	5 (0.3)	13 (0.6)	6 (0.3)	9 (0.4)	4 (0.3)
HPV51	567 (2.7)	52 (3.2)	51 (2.5)	63 (2.9)	65 (2.7)	64 (2.6)	48 (2.4)	55 (2.4)	52 (2.2)	72 (2.9)	45 (2.9)
HPV52	1378 (6.5)	122 (7.4)	120 (5.9)	119 (5.4)	151 (6.4)	170 (7.0)	124 (6.3)	180 (7.9)	141 (6.1)	160 (6.4)	91 (6.0)
HPV53	432 (2.0)	41 (2.5)	56 (2.8)	46 (2.1)	52 (2.2)	49 (2.0)	39 (2.0)	32 (1.4)	44 (1.9)	51 (2.0)	22 (1.4)
HPV56	274 (1.3)	23 (1.4)	16 (0.8)	27 (1.2)	30 (1.3)	39 (1.6)	30 (1.5)	32 (1.4)	25 (1.1)	34 (1.4)	18 (1.2)
HPV58	675 (3.2)	77 (4.7)	60 (3.0)	78 (3.5)	79 (3.3)	80 (3.3)	50 (2.5)	76 (3.3)	66 (2.8)	72 (2.9)	37 (2.4)
HPV59	187 (0.9)	13 (0.8)	13 (0.6)	23 (1.0)	14 (0.6)	21 (0.9)	18 (0.9)	15 (0.7)	22 (0.9)	22 (0.9)	26 (1.7)
HPV66	225 (1.1)	26 (1.6)	22 (1.1)	25 (1.1)	24 (1.0)	21 (0.9)	16 (0.8)	19 (0.8)	28 (1.2)	30 (1.2)	14 (0.9)
HPV68	407 (1.9)	25 (1.5)	36 (1.8)	50 (2.3)	60 (2.5)	43 (1.8)	34 (1.7)	45 (2.0)	42 (1.8)	47 (1.9)	25 (1.6)

HPV, human papillomavirus; CT, *Chlamydia trachomatis*.

**Figure 1:**
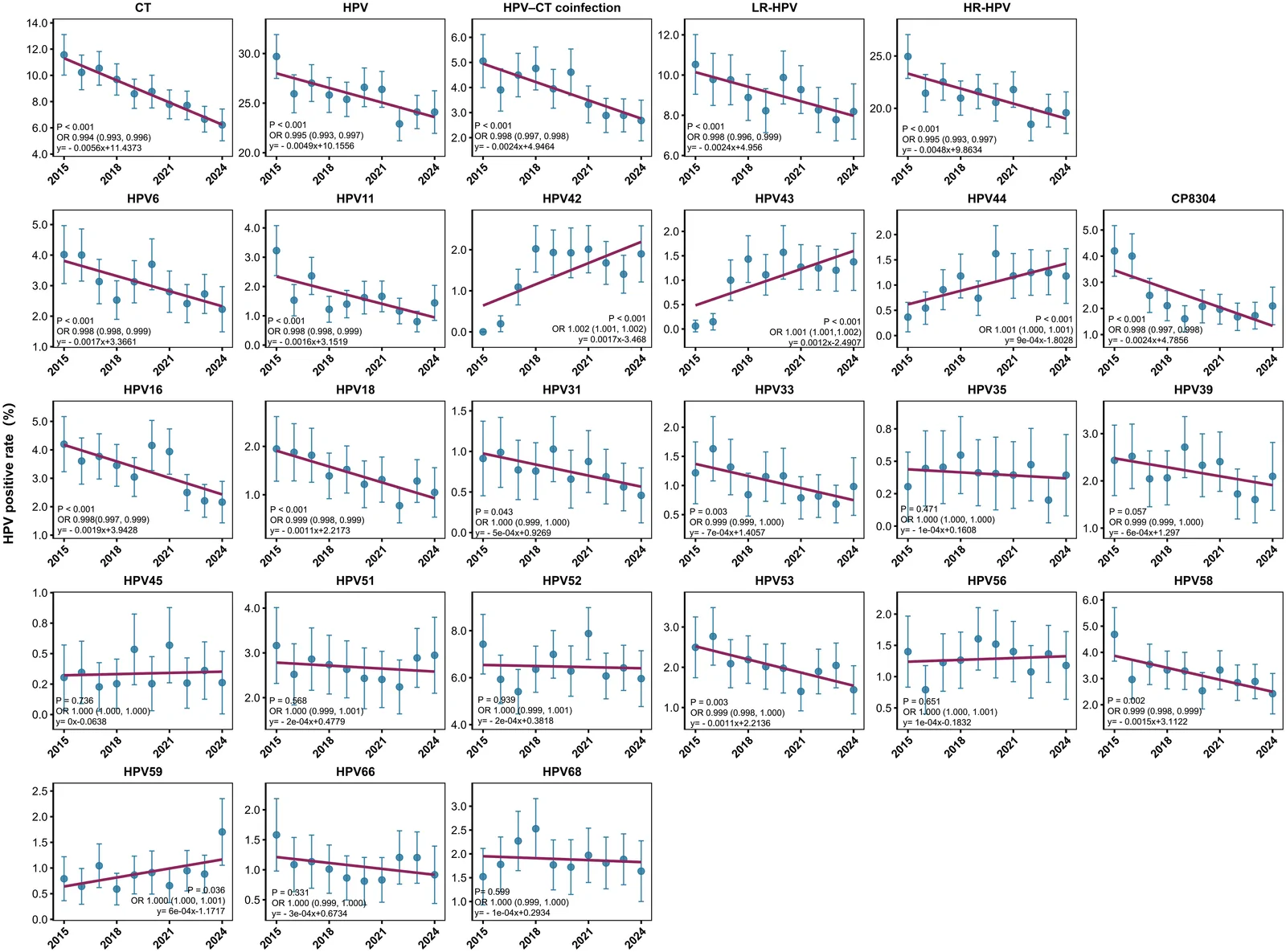
Annual trends in HPV, CT, and HPV–CT coinfection, stratified by HPV genotype, from 2015 to 2024. HPV, human papillomavirus; CT, *Chlamydia trachomatis*; LR-HPV, low-risk HPV; HR-HPV, high-risk HPV.

From 2015 to 2024, the prevalence of HPV, CT, and HPV–CT coinfection decreased significantly, from 29.7% to 24.1%, 11.6% to 6.2%, and 5.1% to 2.7%, respectively (all *p* < 0.001). Declining trends were observed for several HPV genotypes, including HPV16, HPV18, HPV58, HPV6, and HPV11. In contrast, the prevalence of HPV42, HPV43, and HPV44 increased over the study period. HPV52 remained the most prevalent genotype and showed no clear overall decline, with annual prevalence ranging from 5.4% to 7.9% (**Figure 1**; **Table 1**).

### 3.2 Age distribution and epidemiological patterns

**Figure 2:**
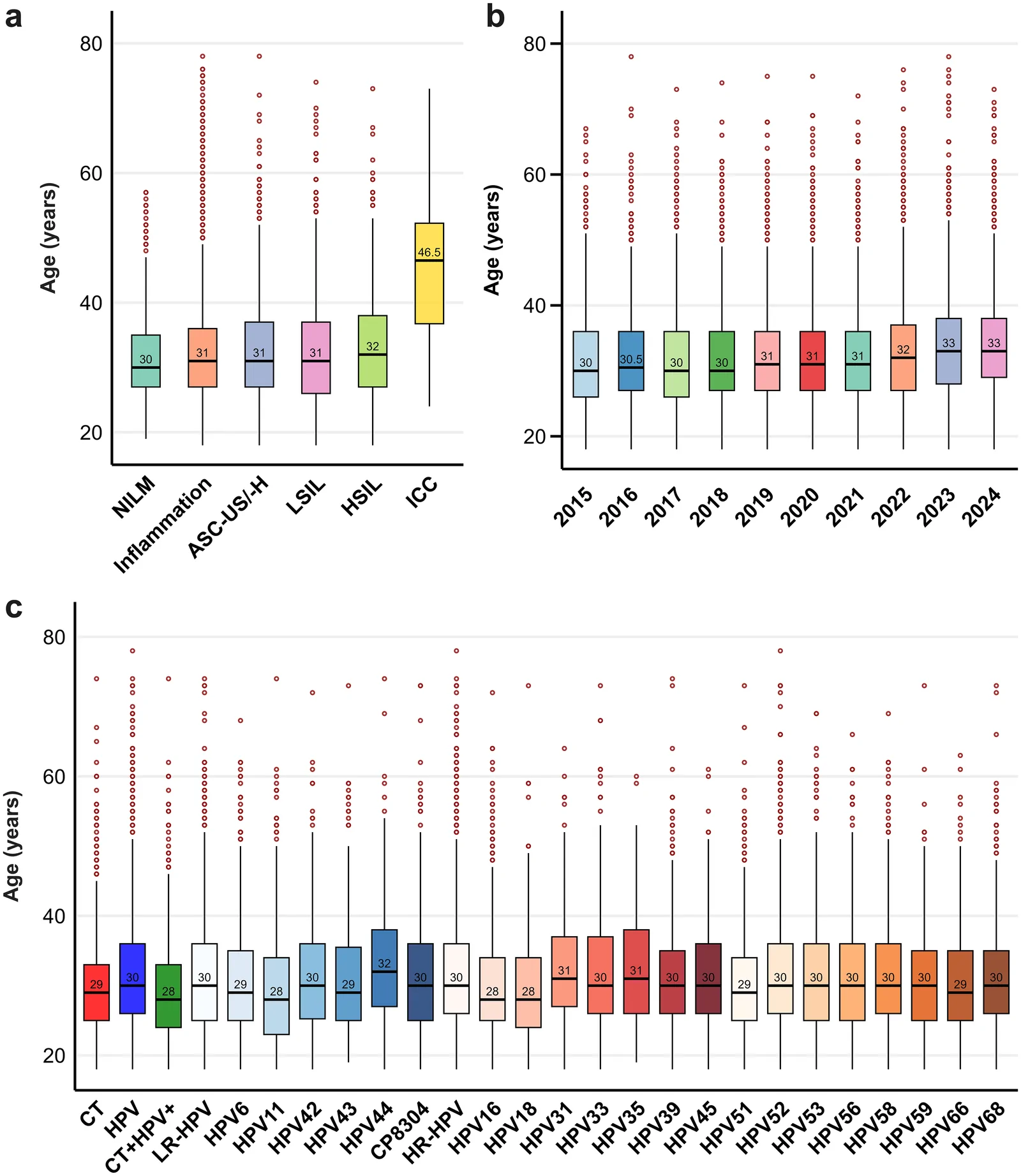
Age distribution by year, cervical lesion category, and infection/genotype status. Boxplots illustrate age distributions of women across **(a)** cytological and histopathological categories, **(b)** calendar years (2015–2024), and **(c)** infection type and HPV genotype. CT, *Chlamydia trachomatis*; HPV, human papillomavirus; LR-HPV, low-risk HPV; HR-HPV, high-risk HPV; NILM, negative for intraepithelial lesion or malignancy; ASC-US/-H, atypical squamous cells of undetermined significance or cannot exclude HSIL; LSIL, low-grade squamous intraepithelial lesion; HSIL, high-grade squamous intraepithelial lesion; ICC, invasive cervical carcinoma; IQR, interquartile range.

The median age of participants was 31 years (IQR 27–36). Participants with more severe cervical lesions tended to be older, with median ages of 30 years in controls, 31 years in the inflammation, ASC-US/-H, and LSIL groups, 32 years in the HSIL group, and 46.5 years in the ICC group (*p* < 0.001) (**Figure 2a**). Over time, the median age at testing also rose, from 30 years in 2015 to 33 years in 2024 (**Figure 2b**).

CT infection (median age, 29 years; IQR, 25–33) and HPV–CT coinfection (28 years; IQR, 24–33) occurred at younger ages than HPV infection alone (30 years; IQR, 26–36; both *p* < 0.001) (**Figure 2c**). HPV16, HPV18, HPV51, and HPV11 were more common in younger women (median age, 28–29 years), whereas HPV44, HPV31, and HPV35 were detected at relatively older ages (median age, 31–32 years). HPV52 and HPV58 showed intermediate age distributions, with a median age of 30 years.

### 3.3 Distribution of CT, HPV–CT coinfection, and HPV across cervical lesion categories

The prevalence of CT and HPV–CT coinfection varied across cervical lesion categories (**Table 2**). CT prevalence was higher in ASC-US/-H (14.4%) and LSIL (10.5%) than in NILM (4.5%, *p* < 0.001), but lower in HSIL (7.1%) and ICC (4.2%). HPV–CT coinfection showed a similar pattern, peaking in ASC-US/-H (9.4%) and LSIL (9.6%) compared with NILM (2.0%, *p* < 0.001). In contrast, HPV prevalence increased with lesion severity, from 12.4% in NILM to 94.6% in HSIL, and was 66.7% in ICC. Within ICC, HPV prevalence was higher in squamous cell carcinoma than in adenocarcinoma (82.1% vs. 45.0%, *p* = 0.007).

**Table 2:** Distribution of HPV genotypes and CT prevalence across cytological and histopathological categories.

HPV and CT	Cervical lesion status, n (%)					
	NILM (n=982)	Inflammation (n=17362)	ASC-US/-H (n=1081)	LSIL (n=1223)	HSIL (n=579)	ICC (n=48)
CT	44 (4.5)	1488 (8.6)^***^	156 (14.4)^***^	128 (10.5)^***^	41 (7.1)^*^	2 (4.2)
HPV	122 (12.4)	3189 (18.4)^***^	539 (49.9)^***^	1037 (84.8)^***^	548 (94.6)^***^	32 (66.7)^***^
HPV–CT coinfection	20 (2.0)	538 (3.1)	102 (9.4)^***^	117 (9.6)^***^	38 (6.6)^***^	2 (4.2)
LR-HPV	51 (5.2)	1183 (6.8)	182 (16.8)^***^	395 (32.3)^***^	103 (17.8)^***^	1 (2.1)
HPV6	13 (1.3)	393 (2.3)	55 (5.1)^***^	148 (12.1)^***^	37 (6.4)^***^	1 (2.1)
HPV11	5 (0.5)	183 (1.1)	38 (3.5)^***^	96 (7.8)^***^	16 (2.8)^***^	0
HPV42	8 (0.8)	204 (1.2)	31 (2.9)^**^	58 (4.7)^***^	8 (1.4)	1 (2.1)
HPV43	4 (0.4)	141 (0.8)	20 (1.9)^**^	44 (3.6)^***^	18 (3.1)^***^	0
HPV44	8 (0.8)	154 (0.9)	18 (1.7)	31 (2.5)^**^	9 (1.6)	0
CP8304	20 (2.0)	297 (1.7)	47 (4.3)^**^	102 (8.3)^***^	28 (4.8)^**^	0
HR-HPV	90 (9.2)	2499 (14.4)^***^	458 (42.4)^***^	887 (72.5)^***^	522 (90.2)^***^	32 (66.7)^***^
HPV16	9 (0.9)	311 (1.8)	67 (6.2)^***^	128 (10.5)^***^	166 (28.7)^***^	18 (37.5)^***^
HPV18	3 (0.3)	142 (0.8)	25 (2.3)^***^	78 (6.4)^***^	46 (7.9)^***^	6 (12.5)^***^
HPV31	1 (0.1)	83 (0.5)	20 (1.9)^***^	32 (2.6)^***^	29 (5.0)^***^	0
HPV33	5 (0.5)	105 (0.6)	22 (2.0)^**^	50 (4.1)^***^	38 (6.6)^***^	2 (4.2)
HPV35	1 (0.1)	52 (0.3)	3 (0.3)	16 (1.3)^**^	13 (2.2)^***^	1 (2.1)
HPV39	13 (1.3)	259 (1.5)	54 (5.0)^***^	98 (8.0)^***^	39 (6.7)^***^	1 (2.1)
HPV45	1 (0.1)	44 (0.3)	2 (0.2)	14 (1.1)^**^	10 (1.7)^***^	2 (4.2)^***^
HPV51	12 (1.2)	323 (1.9)	51 (4.7)^***^	131 (10.7)^***^	48 (8.3)^***^	2 (4.2)
HPV52	23 (2.3)	747 (4.3)^**^	155 (14.3)^***^	266 (21.7)^***^	184 (31.8)^***^	3 (6.2)
HPV53	10 (1.0)	249 (1.4)	47 (4.3)^***^	99 (8.1)^***^	27 (4.7)^***^	0
HPV56	6 (0.6)	149 (0.9)	27 (2.5)^**^	68 (5.6)^***^	23 (4.0)^***^	1 (2.1)
HPV58	8 (0.8)	344 (2.0)^*^	65 (6.0)^***^	147 (12.0)^***^	110 (19.0)^***^	1 (2.1)
HPV59	2 (0.2)	95 (0.5)	19 (1.8)^***^	46 (3.8)^***^	23 (4.0)^***^	2 (4.2)^**^
HPV66	7 (0.7)	121 (0.7)	18 (1.7)	64 (5.2)^***^	15 (2.6)^**^	0
HPV68	11 (1.1)	228 (1.3)	61 (5.6)^***^	87 (7.1)^***^	20 (3.5)^**^	0

HPV, human papillomavirus; CT, Chlamydia trachomatis; LR-HPV, low-risk HPV; HR-HPV, high-risk HPV; NILM, Negative for Intraepithelial Lesion or Malignancy; ASC-US/-H, atypical squamous cells of undetermined significance or cannot exclude HSIL; LSIL, low-grade squamous intraepithelial lesion; HSIL, high-grade squamous intraepithelial lesion; ICC, invasive cervical carcinoma.

NILM served as the reference category.

*** The significance is at the *p* < 0.001 level.

** The significance is at the *p* < 0.01 level.

* The significance is at the *p* < 0.05 level.

**Figure 3:**
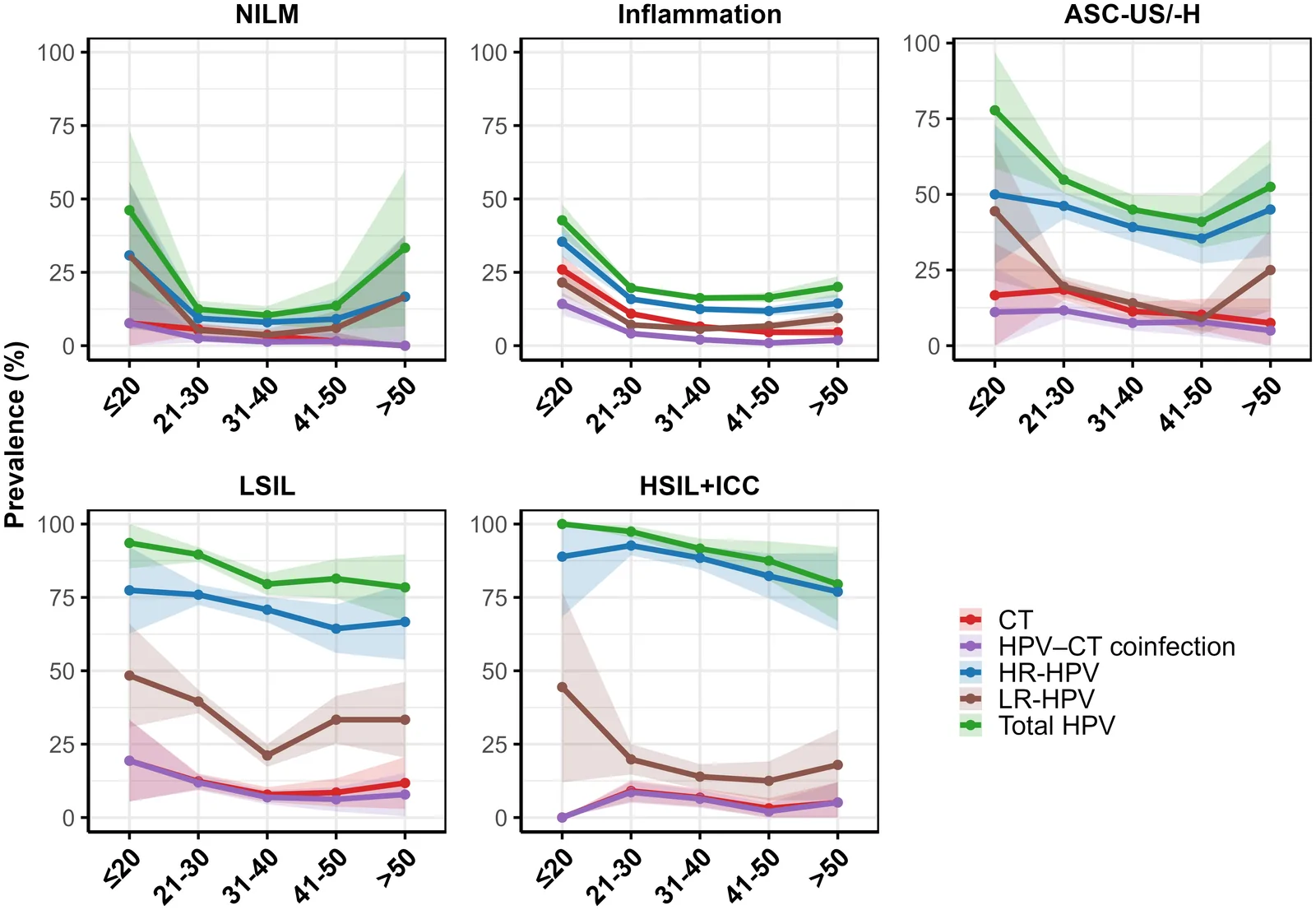
Age-specific prevalence of HPV, CT, and HPV–CT coinfection across cytological and histopathological categories. Line plots show the prevalence of CT, HPV, and HPV–CT coinfection by age group (≤20, 21–30, 31–40, 41–50, and >50 years) stratified by cytological/histopathological diagnosis: NILM, inflammation, ASC-US/-H, LSIL, and HSIL+ICC. Total HPV, HR-HPV, and LR-HPV are displayed separately. CT, *Chlamydia trachomatis*; HPV, human papillomavirus; HR-HPV, high-risk HPV; LR-HPV, low-risk HPV; NILM, negative for intraepithelial lesion or malignancy; ASC-US/-H, atypical squamous cells of undetermined significance or cannot exclude HSIL; LSIL, low-grade squamous intraepithelial lesion; HSIL, high-grade squamous intraepithelial lesion; ICC, invasive cervical carcinoma.

HR-HPV types predominated in high-grade lesions, whereas LR-HPV types were more common in low-grade lesions. HPV16 increased markedly in HSIL and ICC, while HPV52 remained prevalent across lesion categories. Age-stratified analyses (**Figure 3**) showed that CT infection and HPV–CT coinfection were concentrated in younger women, whereas HR-HPV predominated across age groups in HSIL and ICC.

### 3.4 Association between CT positivity, CT load, and HPV status

HPV positivity was higher in CT-positive than CT-negative women (43.9% vs. 23.9%, *χ*^2^ = 354.34, *p* < 0.001) (**Table 3**). This association was evident across diagnostic groups: 45.5% *vs.* 10.9% in NILM, 36.2% *vs.* 16.7% in inflammation, 65.4% *vs.* 47.2% in ASC-US/-H, and 91.4% *vs.* 84.0% in LSIL (all *p* < 0.05). No significant differences were observed in HSIL (92.7% vs. 94.8%, *p* = 0.475) or ICC (100% vs. 65.2%, *p* = 0.546).

**Table 3:** HPV positivity among CT-negative and CT-positive women across diagnostic categories.

Diagnostic category	HPV-positive/total (CT–) (%)	HPV-positive/total (CT+) (%)	*χ* ^2^	*P*
NILM	102/938 (10.9)	20/44 (45.5)	43.07	<0.001
Inflammation	2651/15874 (16.7)	538/1488 (36.2)	342.15	<0.001
ASC-US/-H	437/925 (47.2)	102/156 (65.4)	16.85	<0.001
LSIL	920/1095 (84.0)	117/128 (91.4)	4.29	0.038
HSIL	510/538 (94.8)	38/41 (92.7)	–	0.475
ICC	30/46 (65.2)	2/2 (100)	–	0.546
Total	4650/19416 (23.9)	817/1859 (43.9)	354.34	<0.001

*χ*^2^ test was applied except where expected counts < 5, in which case Fisher’s exact test was used.

HPV, human papillomavirus; CT, *Chlamydia trachomatis*; NILM, negative for intraepithelial lesion or malignancy; ASC-US/-H, atypical squamous cells of undetermined significance/cannot exclude HSIL; LSIL, low-grade squamous intraepithelial lesion; HSIL, high-grade squamous intraepithelial lesion; ICC, invasive cervical carcinoma.

CT bacterial load did not differ by HPV status (median log10 copies/mL: 6.1 vs. 6.2, *p* = 0.14), nor within diagnostic subgroups (all *p* > 0.05) (**Supplementary Figure 1**).

### 3.5 Associations of HPV, CT, and coinfection with cervical lesions

**Figure 4:**
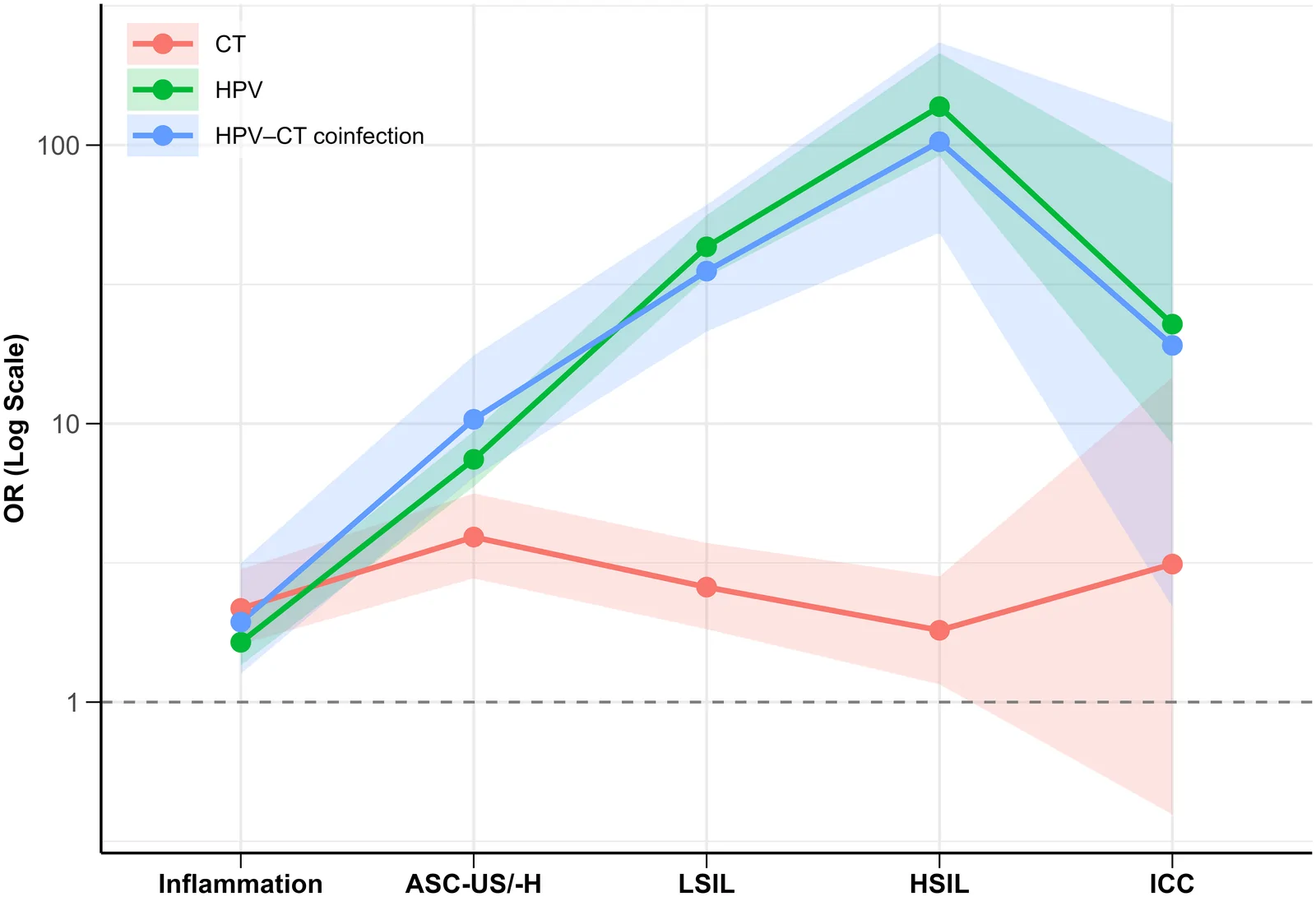
Odds ratios (ORs) for cervical lesions associated with CT infection, HPV infection, and HPV–CT coinfection across diagnostic categories. CT, *Chlamydia trachomatis*; HPV, human papillomavirus; HR-HPV, high-risk HPV; LR-HPV, low-risk HPV; NILM, negative for intraepithelial lesion or malignancy; ASC-US/-H, atypical squamous cells of undetermined significance or cannot exclude HSIL; LSIL, low-grade squamous intraepithelial lesion; HSIL, high-grade squamous intraepithelial lesion; ICC, invasive cervical carcinoma.

Logistic regression analysis showed that CT, HPV, and HPV–CT coinfection were significantly associated with inflammation, ASC-US/-H, and LSIL. For ASC-US/-H, the ORs were 3.9 for CT, 7.4 for HPV, and 10.4 for HPV–CT coinfection (all *p* < 0.001). For LSIL, the corresponding ORs were 2.6, 43.2, and 35.3, respectively (all *p* < 0.001). In HSIL, the associations were strongest for HPV (OR = 137.5, *p* < 0.001) and HPV–CT coinfection (OR = 102.9, *p* < 0.001), whereas CT showed a weaker but significant association (OR = 1.8, *p* = 0.009). In ICC, HPV (OR = 22.8, *p* < 0.001) and HPV–CT coinfection (OR = 19.1, *p* = 0.002) remained significantly associated with disease, whereas CT was not (OR = 3.1, *p* = 0.200). These associations are illustrated in **Figure 4**.

### 3.6 Methylation profiles across study groups

**Figure 5:**
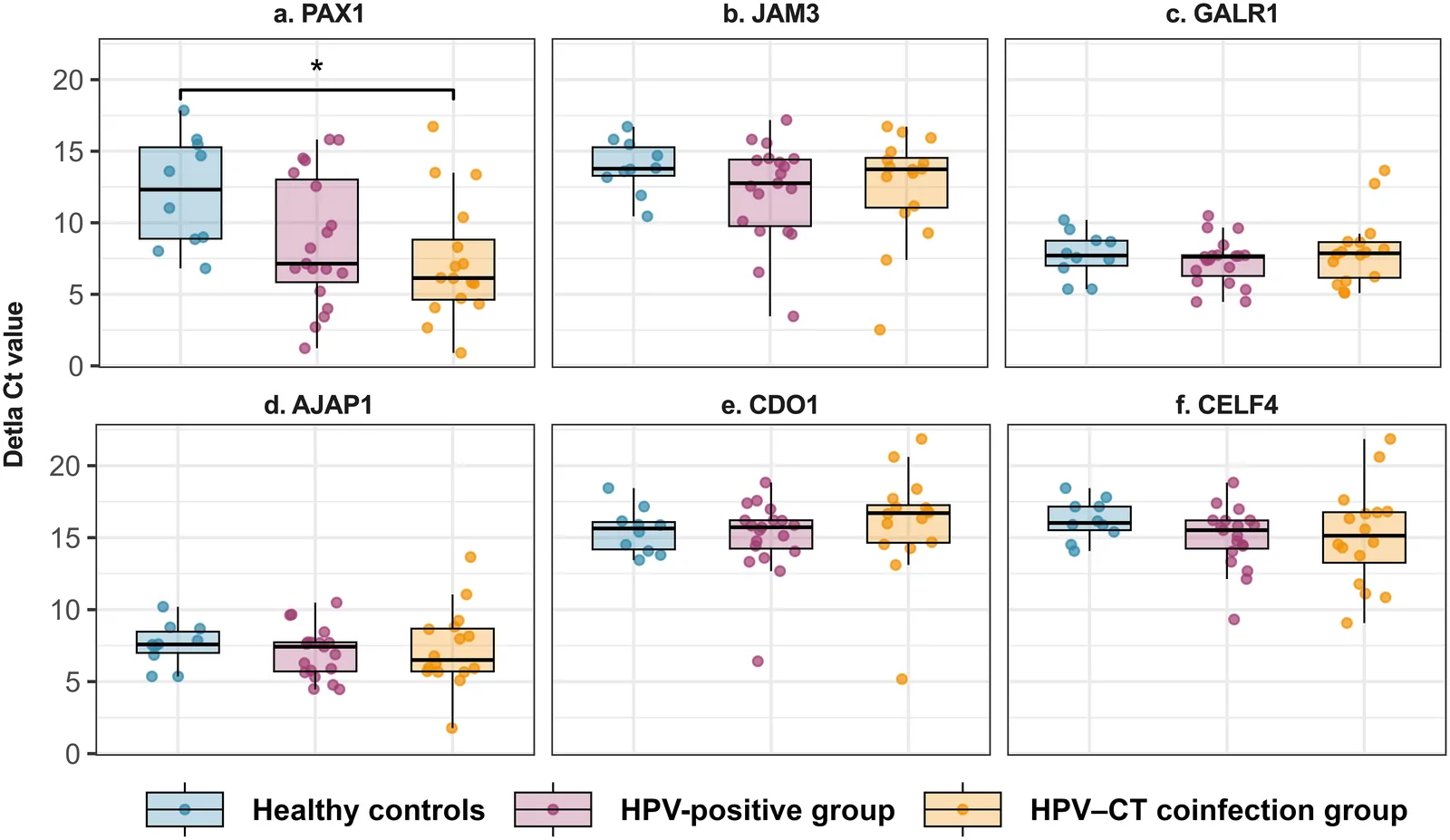
Methylation marker profiles across healthy controls, HPV-positive, and HPV–CT coinfected women. Boxplots show DCt values of PAX1, JAM3, GALR1, AJAP1, CDO1, and CELF4 across three groups: healthy controls (blue), HPV infection alone (pink), and HPV–CT coinfection (orange). PAX1, paired box 1; JAM3, junctional adhesion molecule 3; GALR1, galanin receptor 1; AJAP1, adherens junctions-associated protein 1; CDO1, cysteine dioxygenase type 1; CELF4, CUGBP Elav-like family member 4; HPV, human papillomavirus; CT, Chlamydia trachomatis.

Methylation analysis of 45 cervical samples identified PAX1 as the only marker showing significant intergroup variation (*p* = 0.028) (**Figure 5a**). The mean ΔCt values were 12.11 ± 3.85 in healthy controls, 8.66 ± 4.58 in the HPV-positive group, and 7.31 ± 4.24 in the HPV–CT coinfection group. *Post-hoc* pairwise comparisons showed that the HPV–CT coinfection group had significantly lower PAX1 ΔCt values than healthy controls (*p* = 0.023), whereas no significant differences were observed between healthy controls and the HPV-positive group (*p* = 0.113) or between the HPV-positive and HPV–CT coinfection groups (*p* = 0.630). No significant intergroup differences were observed for JAM3, GALR1, AJAP1, CDO1, or CELF4 (all *p* > 0.05) (**Figures 5b–f**).

## 4 Discussion

In this ten-year retrospective cohort, CT infection and HPV–CT coinfection were more common in younger women and were observed mainly in women with early cervical abnormalities. HPV positivity was also higher among CT-positive women. By comparison, the association between CT and advanced cervical lesions was much weaker, and CT coinfection was not associated with additional methylation changes beyond those observed with HPV infection alone. Overall, HPV showed substantially stronger associations with advanced cervical lesions than CT (Ji et al., 2019; Bray et al., 2024; Lu et al., 2024).

HPV52, 16, and 58 were the predominant high-risk genotypes in our cohort, consistent with previous studies in Chinese women (Shen et al., 2023; Yang et al., 2023; Ding et al., 2025), whereas HPV18 was less frequent than reported in Western populations (de Sanjose et al., 2007; Zhu et al., 2021; Yu et al., 2022). The nonavalent vaccine covers HPV31, 33, 45, 52, and 58 in addition to HPV16/18 and HPV6/11, making this regional genotype distribution relevant to vaccine coverage. Several vaccine-covered genotypes declined during the study period, whereas HPV52 remained common and HPV42, HPV43, and HPV44 increased.

HPV showed a U-shaped age distribution, with peaks in younger women and those aged >50 years, consistent with previous reports (Centre IIHI, 2023; de Sanjose et al., 2007; Ding et al., 2025). CT infection and HPV–CT coinfection were concentrated in younger women. Genotype-specific analysis also showed that HPV16/18 were more frequent in younger women, whereas HPV35/44 were more common in older women (de Sanjose et al., 2007).

Whether CT promotes HPV acquisition, persistence, or lesion progression remains debated. In our cohort, HPV positivity was higher in CT-positive women, whereas CT bacterial load was not associated with HPV status or lesion severity. Previous studies suggest that CT may facilitate HPV persistence through epithelial disruption, immune modulation, and impaired antigen presentation (Silva et al., 2014; Zhu et al., 2016; Ji et al., 2019), although other studies have found no independent association (Safaeian et al., 2010). In our data, the association of CT was most evident in ASC-US/-H and LSIL and was considerably weaker in HSIL and ICC.

HPV positivity was lower in adenocarcinoma (ADC) than in squamous cell carcinoma (SCC), consistent with previous large-scale studies reporting HPV detection rates of nearly 90% in SCC but approximately 70–75% in ADC (Clifford et al., 2003; de Sanjose et al., 2010; Pirog et al., 2014). CT was rarely detected in SCC and was absent in ADC in our cohort.

HPV infection is associated with host DNA methylation alterations, and PAX1 methylation has been proposed as a marker for CIN2+/CIN3+ triage (Lai et al., 2008; Kong et al., 2015; Chen et al., 2024). In our exploratory analysis, PAX1 differed among the three study groups, but no significant difference was observed between HPV infection alone and HPV–CT coinfection. The other five markers showed no intergroup differences. Thus, within the limitations of this small substudy, we found no evidence that concurrent CT infection was associated with additional methylation changes beyond those seen with HPV infection alone.

This study has several limitations. First, the retrospective single-center design and predominantly outpatient population may limit generalizability. Second, the number of invasive cervical cancer cases, particularly adenocarcinomas, was limited, and individual HPV vaccination and CT treatment histories were incompletely available. Third, single-time-point HPV and CT testing and the lack of repeated measurements and longitudinal follow-up limited assessment of persistent infection, dose–response relationships, and temporal dynamics. Finally, the methylation substudy had a modest sample size and a restricted marker panel that excluded widely validated loci such as FAM19A4 and EPB41L3 (Molano et al., 2024). As an observational study, causal and temporal relationships among CT infection, HPV infection, DNA methylation alterations, and cervical lesion progression cannot be established.

## 5 Conclusion

CT infection and HPV–CT coinfection were more common in younger women and were observed mainly in women with low-grade cervical abnormalities. CT-positive women also had a higher prevalence of HPV infection. In contrast, HPV showed much stronger associations with advanced cervical lesions, and CT coinfection was not associated with additional methylation changes beyond those observed with HPV infection alone.

## Data Availability

The raw data supporting the conclusions of this article will be made available by the authors, without undue reservation.
