# Supplementary material for: Clinical significance of Chlamydia trachomatis and HPV coinfection across cervical lesion severity: a 10-year retrospective study in Southern China
Source: Front Cell Infect Microbiol. 2026 Sep 11;16:1832343. doi: 10.3389/fcimb.2026.1832343 (PMC13612179; doi:10.3389/fcimb.2026.1832343)
Supplement: Supplementary file 1 [file DataSheet1.docx]

Supplementary Material

# 1 Supplementary Figures and Tables

## 1.1 Supplementary tables

**Supplementary Table S1.** Distribution of single and multiple HPV infections across cervical cytological and histopathological categories

| HPV infection | Overall (n=21275) | Cervical lesion status, n (%) | | | | | |
| --- | --- | --- | --- | --- | --- | --- | --- |
|  |  | NILM (n=982) | Inflammation (n=17362) | ASC-US/-H (n=1081) | LSIL (n=1223) | HSIL (n=579) | ICC (n=48) |
| Single infection | 3628 (17.1) | 88 (9) | 2265 (13) ^***^ | 338 (31.3) ^***^ | 587 (48) ^***^ | 325 (56.1) ^***^ | 25 (52.1) ^***^ |
| LR-HPV | 907(4.3) | 30 (3.1) | 651 (3.7) | 74 (6.8) ^***^ | 132 (10.8) ^***^ | 20 (3.5) | 0 (0) |
| HR-HPV | 2721(12.8) | 58 (5.9) | 1614 (9.3) ^***^ | 264 (24.4) ^***^ | 455 (37.2) ^***^ | 305 (52.7) ^***^ | 25 (52.1) ^***^ |
| Multiple infection | 1835(8.6) | 34 (3.5) | 921 (5.3) ^*^ | 201 (18.6) ^***^ | 450 (36.8) ^***^ | 222 (38.3) ^***^ | 7 (14.6) ^**^ |
| Double | 1152 ( 5.4) | 26 (2.6) | 585 (3.4) | 134 (12.4) ^***^ | 262 (21.4) ^***^ | 140 (24.2) ^***^ | 5 (10.4) ^*^ |
| Triple | 418 ( 2.0) | 3 (0.3) | 215 (1.2) ^*^ | 40 (3.7) ^***^ | 111 (9.1) ^***^ | 47 (8.1) ^***^ | 2 (4.2) ^*^ |
| Quadruple or more | 265 ( 1.2) | 5 (0.5) | 121 (0.7) | 27 (2.5) ^***^ | 77 (6.3) ^***^ | 35 (6) ^***^ | 0 (0) |

HPV, human papillomavirus; LR-HPV, Low-risk HPV; HR-HPV, High-risk HPV; NILM, Negative for Intraepithelial Lesion or Malignancy; ASC-US/-H, atypical squamous cells of undetermined significance or cannot exclude HSIL; LSIL, low-grade squamous lesion; HSIL, high-grade squamous lesion; ICC, invasive cervical carcinoma.

NILM served as the reference category.

***The significance is at the p < 0.001 level.

**The significance is at the p < 0.01 level.

*The significance is at the p < 0.05 level.

| **Supplementary Table S2.** HPV genotype and *Chlamydia trachomatis* prevalence in invasive cervical carcinoma subtypes | | |
| --- | --- | --- |
| HPV and CT | Invasive cervical carcinoma, n (%) | |
|  | Squamous cell carcinoma (n=28) | Adenocarcinoma (n=20) |
| CT | 2 (7.1) | 0 |
| HPV | 23 (82.1) | 9 (45.0) |
| HPV-CT coinfection | 2 (7.1) | 0 |
| LR-HPV | 1 (3.6) | 0 |
| HPV6 | 1 (3.6) | 0 |
| HPV11 | 0 | 0 |
| HPV42 | 1 (3.6) | 0 |
| HPV43 | 0 | 0 |
| HPV44 | 0 | 0 |
| CP8304 | 0 | 0 |
| HR-HPV | 23 (82.1) | 9 (45.0) |
| HPV16 | 14 (50.0) | 4 (20.0) |
| HPV18 | 3 (10.7) | 3 (15.0) |
| HPV31 | 0 | 0 |
| HPV33 | 2 (7.1) | 0 |
| HPV35 | 1 (3.6) | 0 |
| HPV39 | 1 (3.6) | 0 |
| HPV45 | 0 | 2 (10.0) |
| HPV51 | 1 (3.6) | 1 (5.0) |
| HPV52 | 2 (7.1) | 1 (5.0) |
| HPV53 | 0 | 0 |

## 1.2 Supplementary Figure

**Supplementary Figure S1.** Comparison of CT bacterial load between HPV-positive and HPV-negative women. Bacterial load was measured as log₁₀ copies/mL. No significant differences were observed between HPV-positive and HPV-negative women overall (median 6.1 vs. 6.2, p = 0.14) or within diagnostic subgroups, including NILM (p = 0.46), inflammation (p = 0.79), ASC-US/-H (p = 0.066), LSIL (p = 0.93), and HSIL/ICC (p = 0.62). CT, Chlamydia trachomatis; HPV, human papillomavirus; NILM, negative for intraepithelial lesion or malignancy; ASC-US/-H, atypical squamous cells of undetermined significance or cannot exclude HSIL; LSIL, low-grade squamous intraepithelial lesion; HSIL, high-grade squamous intraepithelial lesion; ICC, invasive cervical carcinoma.

**Supplementary Figure 1.** The figure legends are required to have the same font as the main text, 12 point normal Times New Roman, single spaced. Please use a single paragraph for each legend and prepare the figures keeping in mind the PDF layout.
